# Comparative study on the effects of different polishing methods on tooth surface microstructure and roughness following initial periodontal treatment

**DOI:** 10.1002/cre2.851

**Published:** 2024-01-31

**Authors:** Jingjue Gong, Xin Huang, Shuang Yuan

**Affiliations:** ^1^ Dental Department Shanghai Jing‐an Dental Clinic Shanghai China

**Keywords:** polishing, polishing disc, polishing strip, silica onegloss

## Abstract

**Objective:**

To investigate the effects of different polishing methods on the surface microstructure and roughness of teeth following initial periodontal treatment.

**Methods:**

Teeth were divided into three groups (silica onegloss, polishing disc, and polishing strip) in vitro. Tooth surface microstructure was characterized via scanning electron microscopy. Roughness was measured by profilometry and laser scanning confocal microscopy (LSCM). According to the in vitro results, one group was chosen for further clinical trials. Effects of the chosen polishing method on patient plaque control and satisfaction were assessed via plaque staining and visual analog scale (VAS).

**Results:**

Electron microscopy results revealed that the polishing disc group had smoother roots and crowns than did the other two groups. Roughness analysis revealed that the crown roughness with the polishing disc and silica onegloss was lower, and the root roughness with the polishing disc was the lowest (*p* < .05) The LSCM results showed that the pigment deposition depth with the polishing disc and silicon onegloss in the crowns and roots were significantly lower than those with the other two methods (*p* = .000). The polishing effect of the polishing disc was best among the three groups. Clinical trials were performed to verify the polishing disc effects. Plaque staining results showed that the decrease in plaque in the polishing disc group was greater than that of the rubber cup group (*p* = .020), which was similar to the results of LSCM. The VAS results showed that the polishing disc made teeth feel smoother, similar to the results of the roughness detection, and the procedure was more comfortable (*p* < .05). However, there was no significant difference between the two groups regarding foreign body sensation during pronunciation (*p* = .178).

**Conclusion:**

Combining in vitro and in vivo evaluations, the use of a polishing disc following periodontal treatment yielded superior polishing effects on teeth and was better accepted by patients.

## 1 INTRODUCTION

Periodontitis is one of the most common oral diseases. According to the results of the fourth national oral health epidemiological survey in China, the detection rates of dental calculus and gingival bleeding in 35–44‐year‐old people were 96.7% and 87.4%, respectively. Thus, periodontal health must be further improved. Periodontitis is essentially a mixed bacterial infection that destroys periodontal support tissues (Teles et al., 2022), and untreated periodontitis often leads to tooth displacement, loosening, and loss (Cobb & Sottosanti, 2021); therefore, the treatment of periodontitis has received increasing attention.

Periodontitis is generally considered an infectious disease caused by microorganisms, and the most important cause of this disease is plaque with its attached bacteria (Cao, 2018). Therefore, a comprehensive understanding of plaque formation and biological characteristics is essential for the control and prevention of periodontal disease. To date, plaques are generally divided into supragingival and subgingival plaques according to their distribution. Supragingival plaque is generally distributed in the enamel or gingival margin above the cemento–enamel junction, and it mainly includes Gram‐positive cocci and bacilli. Subgingival plaque is generally distributed in the cementum below the cemento–enamel junction in the periodontal pocket, and it mainly contains gram‐negative bacilli (Susin et al., 2023). In the same oral environment, bacteria inside plaque biofilms are 1000–1500 times more resistant to antibiotics than bacteria outside plaque biofilms (Siawasch et al., 2022). Within dental plaque, there are multiple microenvironments with different pH values and concentrations, enabling various species with different metabolic needs to survive in them and increasing the diversity of bacterial populations (Albeshri & Greenstein, 2022; Scannapieco & Dongari‐Bagtzoglou, 2021). This diversity of species makes it increasingly difficult to control mixed infections caused by dental plaque. Therefore, controlling plaque formation and proliferation has important implications for treating periodontitis.

To date, a common method for removing and controlling plaque involves a basic treatment with supportive therapy for periodontitis. Periodontal scaling and curettage of supragingival and subgingival areas and sandblasting are the most important procedures (Lin et al., 2023); however, there are many reports suggesting that basic treatment removes plaque and increases the surface roughness of teeth (Benfenati et al., 1987; Bühler et al., 2016; Caygur et al., 2017). In clinical work, polishing in periodontitis cases after scaling and curettage includes silicon onegloss and rubber cup polishing, which improves the surface smoothness of teeth, reduces the adhesion of bacteria and food residue, and slows the rate of reformation of dental calculus (Oda et al., 2004). However, the effect of these methods remains somewhat controversial. Previous studies, such as that conducted by Huang Jianfeng, have shown that the reduction in the surface roughness of teeth polished in vitro polished by sandblasting is more obvious than that of in vitro by a rubber cup (Jianfeng, 2016). Yuanli et al. (2018) have noted that different polishing methods have different advantages and disadvantages in terms of four aspects, namely, the plaque index, gingival index, bleeding index, and pigment index, and the method should be selected according to patient pigment deposition, gingival bleeding, and plaque deposition. Chowdhary and Mohan (2018) compared three polishing methods—brush, rubber cup, and air scrub—by dividing 60 periodontitis‐excised teeth from patients with periodontitis into different enamel and cementum samples. It was concluded that rubber cup polishing has a good polishing effect on both gingival and subgingival surfaces. Although there are many polishing methods, such as rubber cups, hair brushes, and silicon onegloss, it is not clear which polishing method has the best influence on the surface microstructure and roughness of teeth.

In view of these findings, we focused on the effects of different polishing methods on the surface microstructure and roughness of periodontitis patients' teeth after basic treatment in vitro in this study. The effects of different polishing methods were evaluated via in vitro experiments, which contained electron microscopy, profilometry, and laser scanning confocal microscopy (LSCM). According to the in vitro results, one group was chosen for further clinical trials. Plaque staining and visual analog scale (VAS) analysis were used to verify the effects of the chosen polishing method on patient plaque control and self‐perception and the results of profilometry and LSCM.

The experimental data and clinical references are provided for clinicians to select different polishing methods.

## 2 MATERIALS AND METHODS

### 2.1 In vitro experiment

#### 2.1.1 Collection of in vitro teeth

Quantity, grouping, and source: A total of 40 teeth with periodontitis were extracted from the Jingan Dental Defence Institute from July 2020 to December 2020. The ages of the patients ranged from 35 to 55 years old, and every patient has signed an informed consent form. Forty teeth were randomly and evenly divided into four groups according to the different polishing instruments used: no polishing agent (control group), polishing disc (L507/522/519/521,SHOFU, Japan), polishing strip (PN L525/L526,SHOFU, Japan), and silicon onegloss (DR001,SHOFU, Japan). After extraction, the teeth were stored in 5% hydrogen peroxide water for 2 h. Then, the sections were rinsed for 2 min and dried for analysis. All the teeth were subjected to scaling and blasting before polishing.

Inclusion criteria: tooth mobility of III°, intact teeth, premolars or molars.

Exclusion criteria:

1. Inclusion of prostheses
2. Fracture cracks
3. X‐ray film showing signs of outer root absorption
4. Caries
5. Wear and tear

#### 2.1.2 Surface standardization and polishing of teeth in vitro

The mesial and distal surfaces of each tooth were selected as the working area; the working area included 5 mm of the crown to 5 mm of the root with the cemento–enamel junction serving as the midline. Every tooth was subjected to ultrasonic scaling on the whole working area and manual scaling on the root for 30 s. An ultrasonic scaler (suprasson p5 newtron; SATELEC) was used for ultrasonic scaling. The front edge of the work head was parallel to or less than 15° angle to the tooth surface. The roots were curetted with a Gracey curettage device (SC13/14; HU‐Friedy). When removing the root calculus, we scraped the edge of the blade at an angle of approximately 80° to the root surface. Sandblasting was performed using a sandblasting instrument (prophy‐mate; NSK) with polishing powder (AIR N GO; SATELEC), which contained sodium bicarbonate, hydrophobic silica, and saccharin sodium. And the particle size of the powder was about 65 μm. The teeth were grouped according to the polishing methods after standardization treatment. The polishing instrument was applied by circular motion for 60 s with a dental handpiece at a low speed of 200 r/min and a pressure of 5 N (in which the polishing disc was used in the order of black, purple, green and red, and each color lasted for 15 s); conversely, the hand‐held polishing strip was applied in a straight line for 60 s at 30 times/min (in the order of black, purple, green and red, and each color lasted for 15 s). After polishing, the teeth were rinsed for 20 s and subsequently dried.

#### 2.1.3 In vitro detection of the tooth surface

We made tooth sections from the mesial and distal surfaces of the teeth for scanning electron microscopy (SEM) and profilometry. The tooth surface roughness was measured using a profilometer (UP‐WLI; Rtec) before and after polishing in the three experimental groups. The copies used in SEM were 2 × 2 × 4 mm from the mesial and distal surfaces of each tooth, and we standardized the size and location of the scanned area in the middle of each copy. Before SEM, the sections were coated with gold through ion sputtering. The microstructures of the dental surfaces and the distributions of debris scratches were observed by SEM (Mira4; Tescan) in high vacuum (10,000×). To evaluate the ability of teeth to resist discoloration after polishing, we placed the teeth in 0.1% rhodamine B solution in a 37°C water bath condition for 24 h and rinsed them with distilled water for 10 s. Grand sections were cut from each tooth and an LSCM (A1; Nikon) was used to observe the staining bands and to measure the depth of the pigmentation bands (×10 objective for photography; excitation light wavelength 561 nm; emission wavelength 600 nm).

$$\text{Profilometer detection index}:\;\text{roughness ratio}=\frac{\text{RA value before polishing}}{\text{RA value after polishing}}.$$

Confocal measurements: band width.

### 2.2 Clinical trials

#### 2.2.1 Study subjects

Quantity, source, and groups: A total of 60 patients with periodontitis were selected from the Jingan District Dental Defence Center from January 2022 to December 2022. The ages of the patients ranged from 35 to 55 years old, and the patients were randomly divided into two groups. The experimental group was polished by the chonsen method, and the control group was polished by Rubber Cup (CR POLISHER PS; SHOFU). The content and informed consent for this study were reviewed and approved by the Jing'an District Ethics Committee.

Inclusion criteria: Patients aged 35–55 years without systemic diseases who had a history of regular dental cleaning in the past 3 years had not received periodontal treatment in the past year and were willing to enter the study.

Exclusion criteria:

1. Patients who did not agree to participate in the study.
2. Patients who had undergone periodontal therapy in the past year.
3. Patients with a history of systemic disease who were not suitable for periodontal therapy.
4. Patients who had not received periodontal treatment in the last 3 years.
5. Patients who could not follow up.

Plaque staining: A small cotton ball was dipped into the plaque stain (Mira‐2‐Ton; Hager & Werken) and gently squeezed it at the gingival papilla to allow the stain to overflow from the cotton ball and evenly distribute it on the buccal and lingual sides of the teeth. The plaque coverage percentage was measured by plaque staining before polishing and at 24 h after polishing in the control group and experimental group.

The positive percentage of plaque staining was calculated as the number of stained dental surfaces/the number of total dental surfaces × 100%.

VAS: Patients were asked to rate their feelings before and after the polishing according to the VAS (Table 1).

**Table 1:** Visual analog scale.

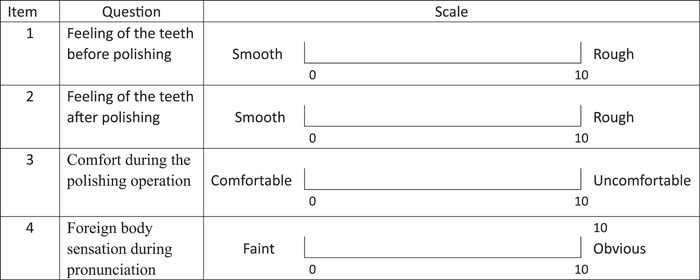

The VAS is the length of the visual line implemented to reflect the feelings of the patient; the scored items include operation comfort, feeling of dental roughness, and foreign body sensation in the mouth when speaking.

**Figure 1:**
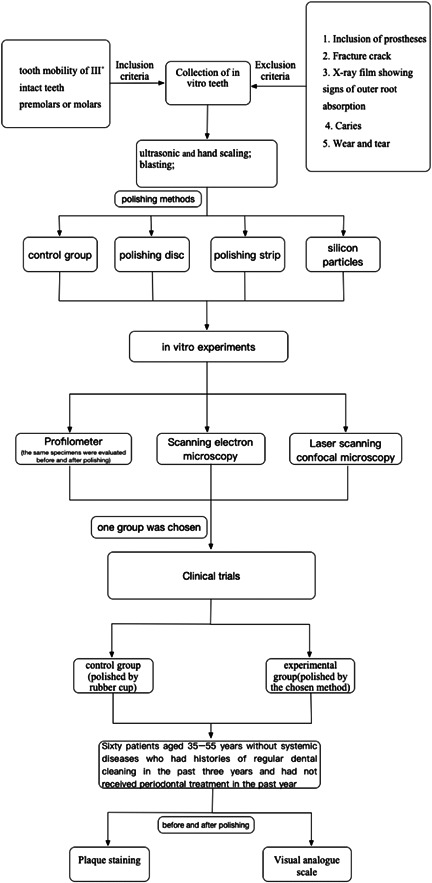
Study design figure.

We have added a study design figure that clearly depicts the study groups in each part (Figure 1).

### 2.3 Statistical analysis

One‐way analysis of variance was used for the roughness test results and the crown results of the LSCM. The root results of LSCM were tested by an independent sample nonparametric test. Paired *T* tests were used for plaque staining and VAS score. A *p* < .05 suggested that there was a significant difference, a *p* < .01 suggested that there was a more reliable difference, and a *p* > .05 suggested that there was no significant difference.

## 3 RESULTS

### 3.1 In vitro experiments

#### 3.1.1 SEM observations

**Figure 2:**
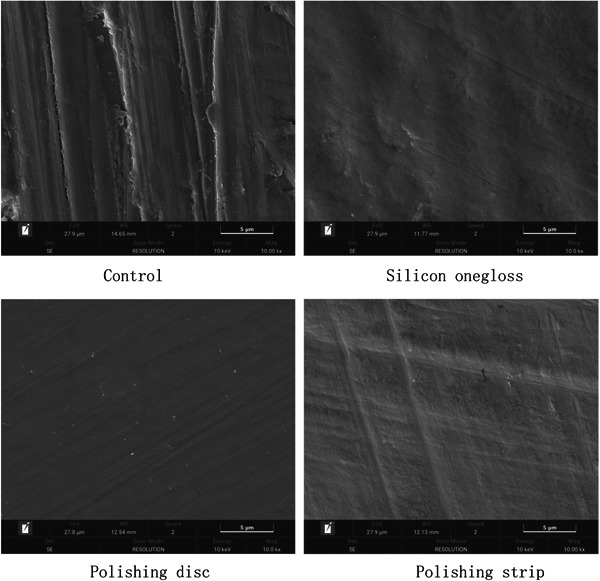
Crown scanning electron microscopy images.

**Figure 3:**
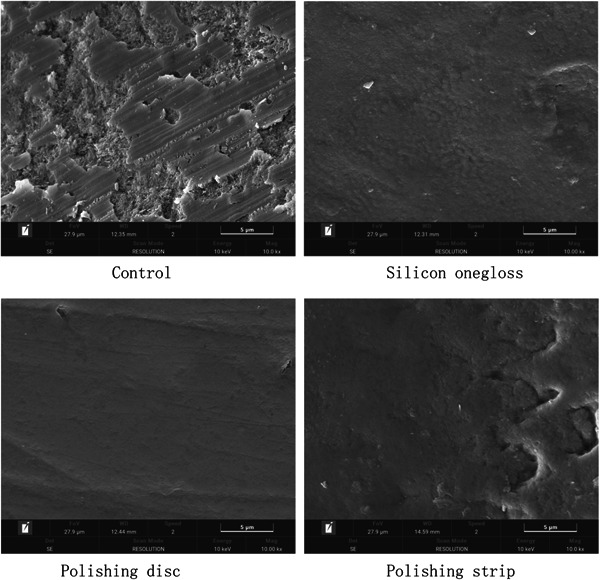
Root scanning electron microscopy images.

The SEM results showed that the crown and root surfaces of the plants in the control group had obvious wear and debris (Figures 2 and 3). There were fewer scratches on the crown or root sides of the silicon onegloss group and polishing disc group, which comfirmed the effectiveness of polishing. In addition, spot‐shaped defects could still be observed on the crown and root sides in the polishing strip group, indicating a weaker polishing effect. According to the electron microscopy observations, the polishing effects of the polishing disc group and the silicon onegloss group were better than that of the polishing strip group.

#### 3.1.2 Roughness measurements

**Figure 4:**
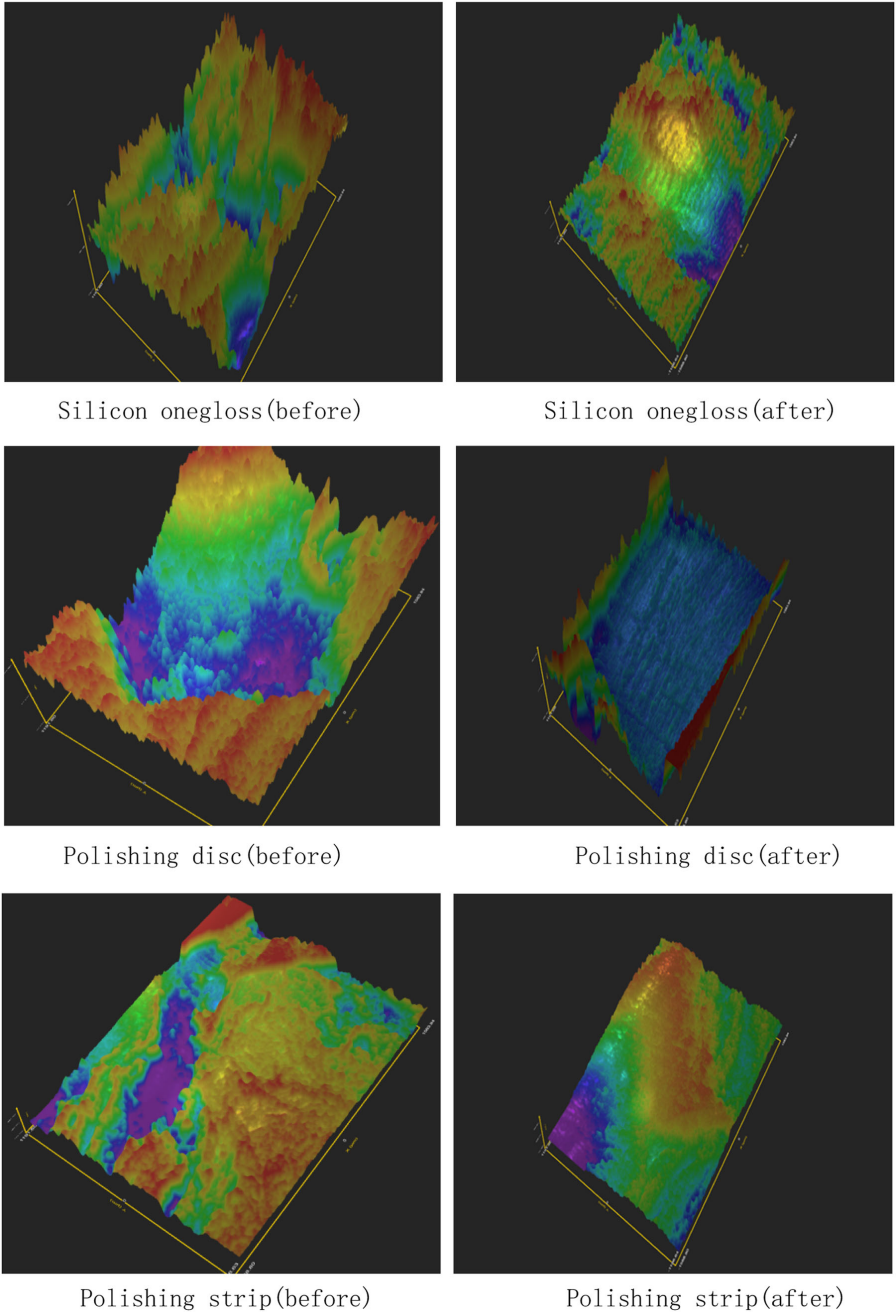
Roughness of the crown.

**Figure 5:**
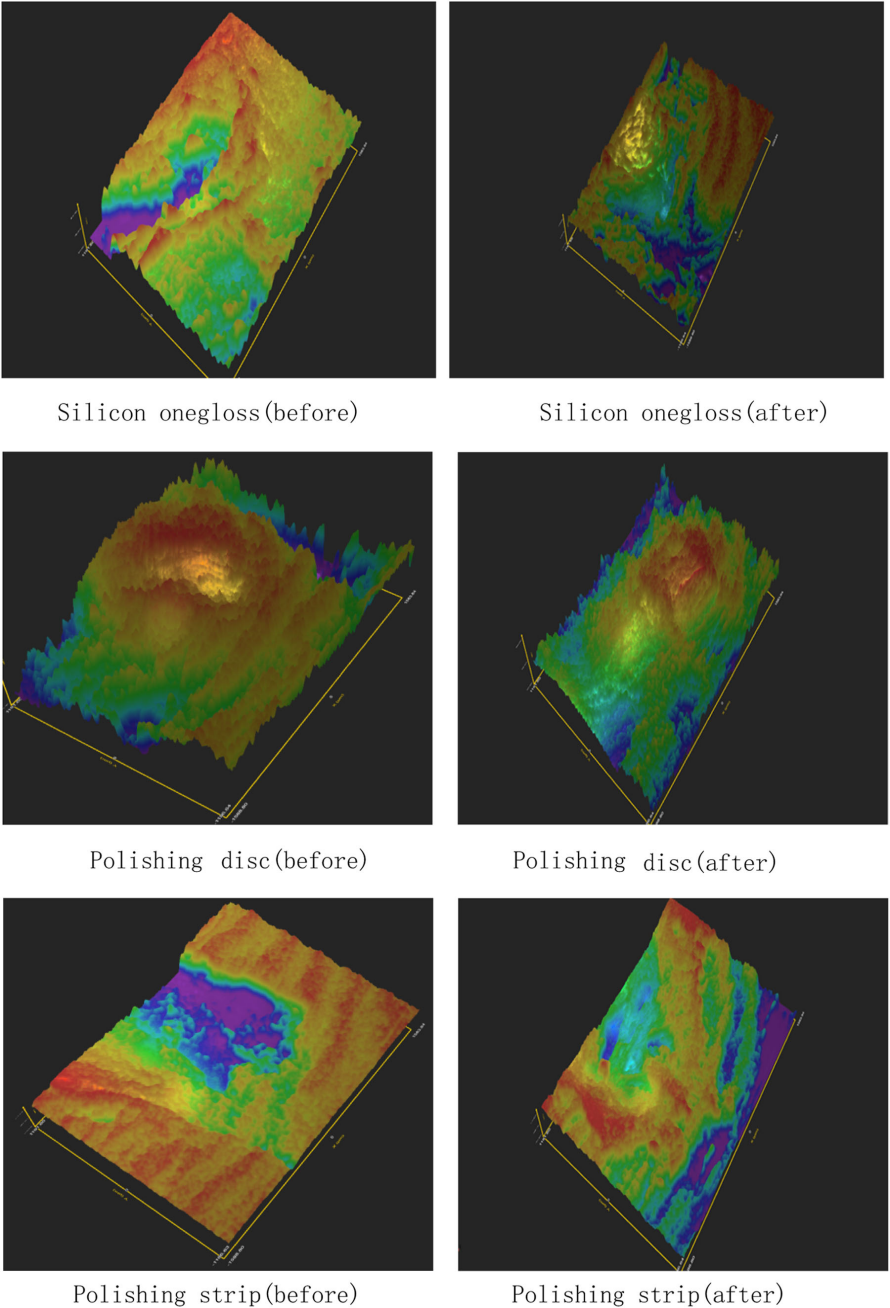
Roughness of the root.

**Figure 6:**
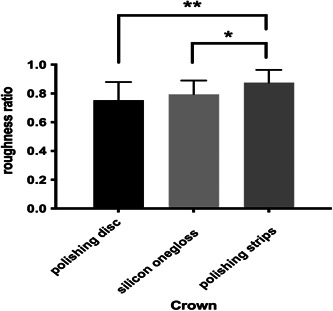
Crown of the profilometer.

**Figure 7:**
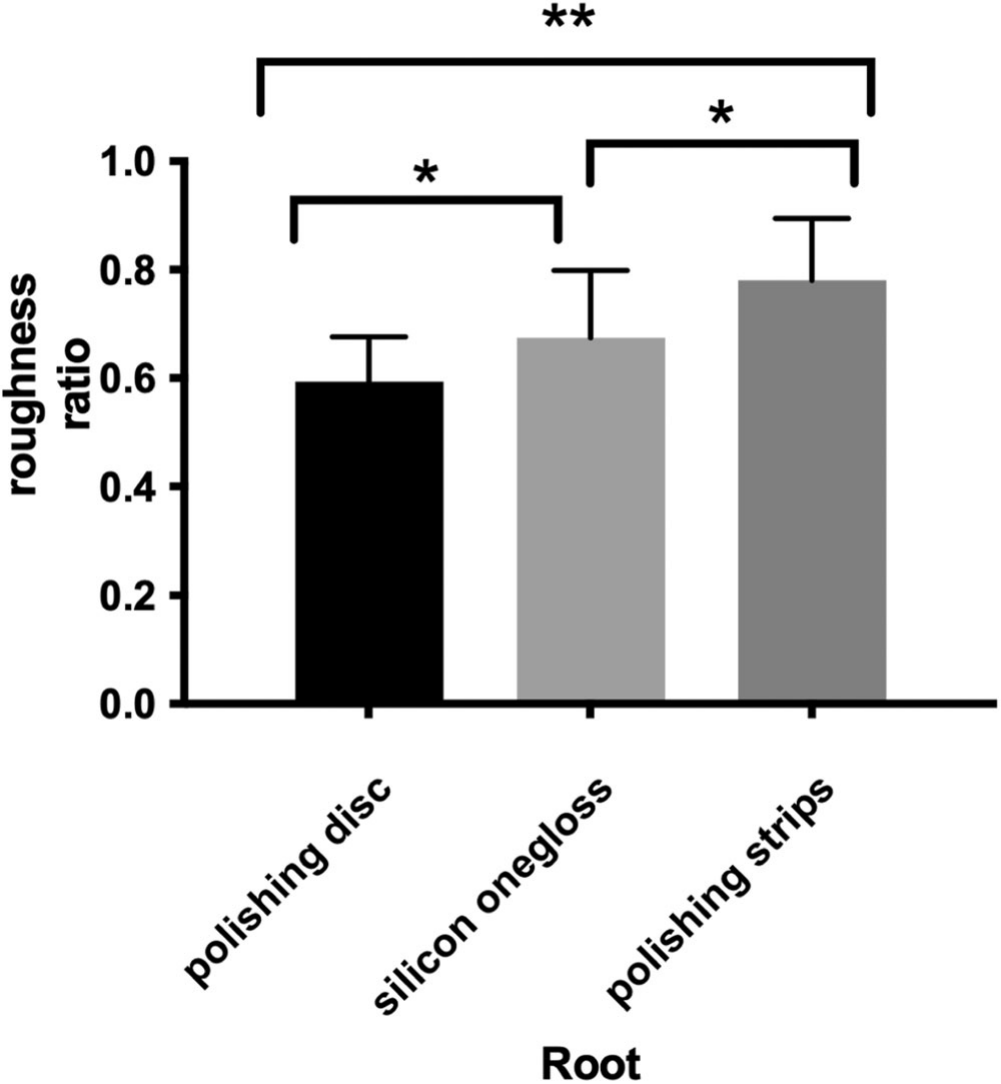
Root of the profilometer.

Figures 4 and 5 show the three‐dimensional (3D) characteristics of the Ra value distribution. The blue area indicates a lower Ra value, while the red area indicates a higher Ra value. We could see more blue areas before polishing and more red areas after polishing. We picked five square areas (1 mm × 1 mm) in each tooth section to measure the Ra value. The results of the relative ratio of roughness detection (roughness after polishing/roughness before polishing) are shown in Tables 2 and 3. The three experimental groups had significantly lower roughness ratios (*p* = .000) than the control group (Tables 4 and 5); additionally, the roughness ratio on the roots was lower than that on the crown in each group (*P* disk = 0.000, *P* silicon = 0.006, *p* strip = 0.016), and the roughness ratios of the silicon onegloss group and polishing disc group were lower than that of the polishing strip group on the crown (*P* silicon and disc = 0.287, *P* silicon and strip = 0.040, *P* disc and strip = 0.003). The roughness ratio of the roots in the polishing disc group was lower than that of the silicon onegloss group, which was lower than that of the polishing strip group (*P* silicon and disc = 0.048, *P* silicon and strip = 0.010, *P* disc and strip = 0.000) (Figures 6 and 7). Table 6 showed that the root roughness ratio were significantly lower the crown roughness ratio in the same group.

**Table 2:** Roughness ratio and statistical detection of roughness changes after polishing the crown.

	Roughness ratio after polishing	Statistical detection
Polishing disc	0.7534 ± 0.1260	0.000*
Silicon onegloss	0.7946 ± 0.0962	0.000*
Polishing strip	0.8754 ± 0.0877	0.000*

*p < 0.05.

**Table 3:** Roughness ratio and statistical detection of roughness changes after polishing the roots.

	Roughness ratio after polishing	Statistical detection of roughness changes
Polishing disc	0.5940 ± 0.0820	0.000*
Silicon onegloss	0.6744 ± 0.1243	0.000*
Polishing strip	0.7806 ± 0.1139	0.000*

*p < 0.05.

**Table 4:** Statistical detection of roughness changes between the different experimental groups.

	Crown
Polishing disc versus silicon onegloss	0.287
Silicon onegloss versus polishing strip	0.040*
Polishing disc versus polishing strip	0.003**

*p < 0.05; **p < 0.01.

**Table 5:** Statistical detection of roughness changes between different experimental groups.

	Root
Polishing disc versus silicon onegloss	0.048*
Silicon onegloss versus polishing strip	0.010*
Polishing disc versus polishing strip	0.000**

*p < 0.05; **p < 0.01.

**Table 6:** Statistical detection of roughness changes between roots and crowns in different experimental groups.

	Crown versus root
Polishing disc	0.000
Silicon onegloss	0.006**
Polishing strip	0.016*

*p < 0.05; **p < 0.01.

#### 3.1.3 LSCM

**Figure 8:**
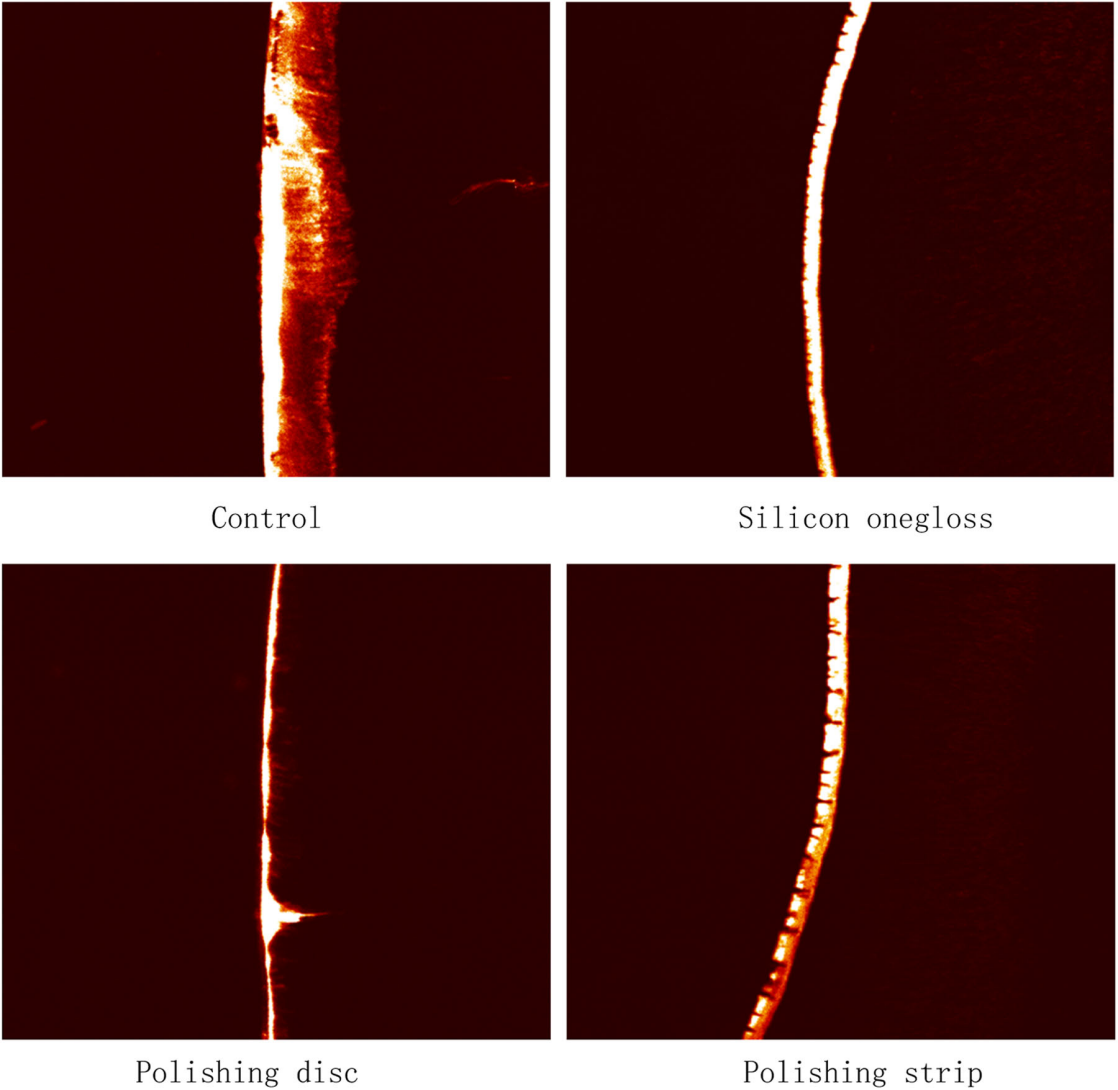
Pigmentation depth of the crown.

**Figure 9:**
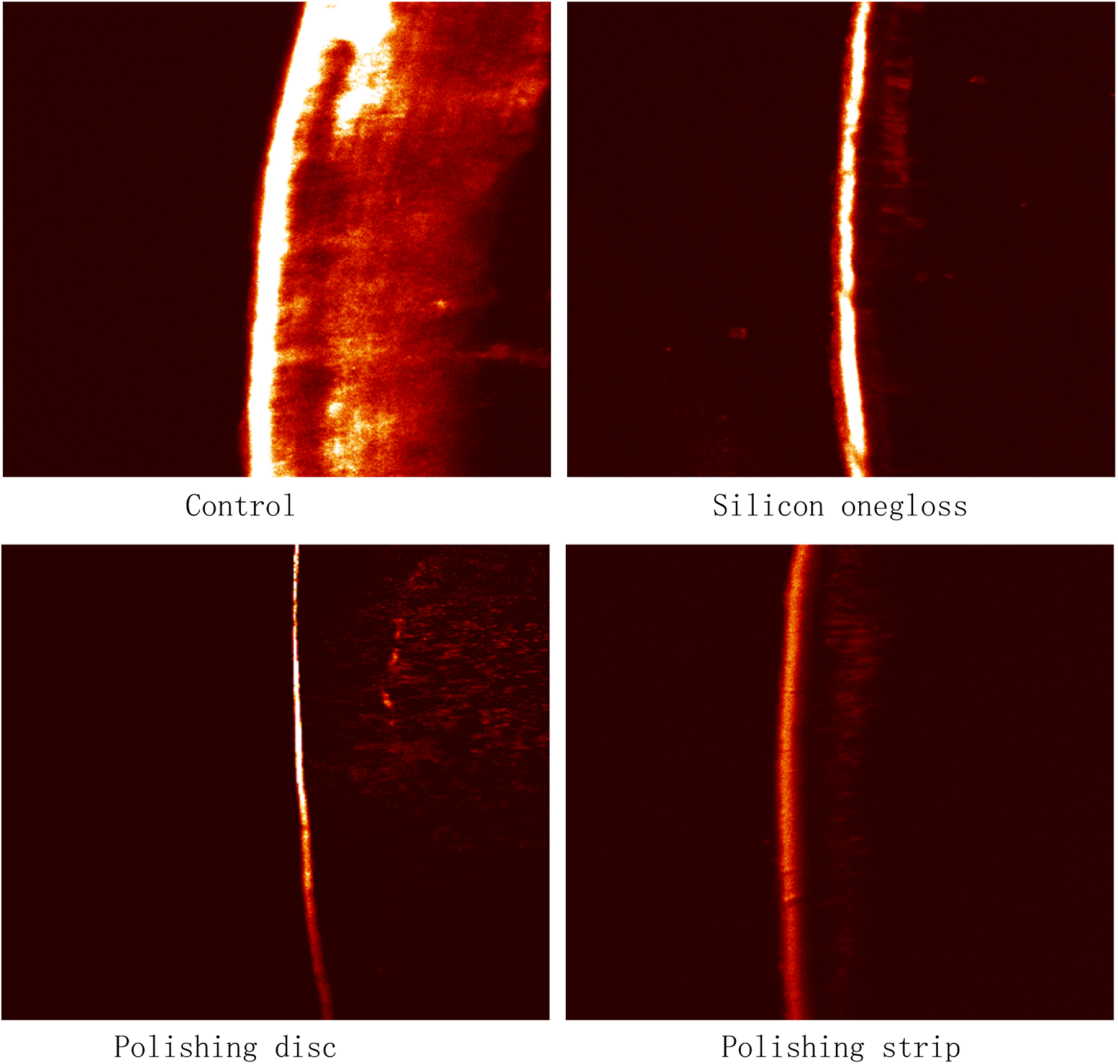
Pigmentation depth of the root.

**Figure 10:**
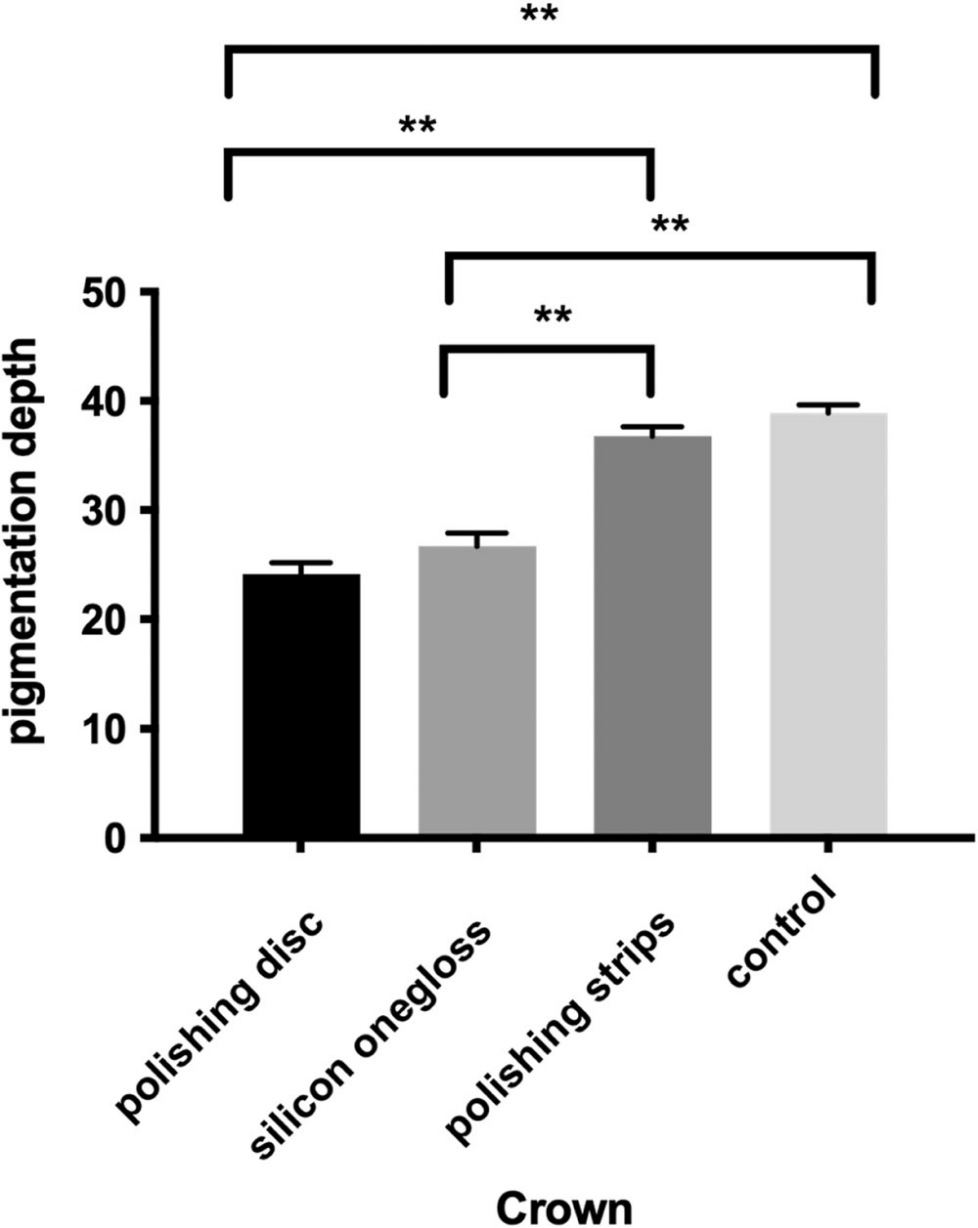
Crowns assessed by laser scanning confocal microscopy.

**Figure 11:**
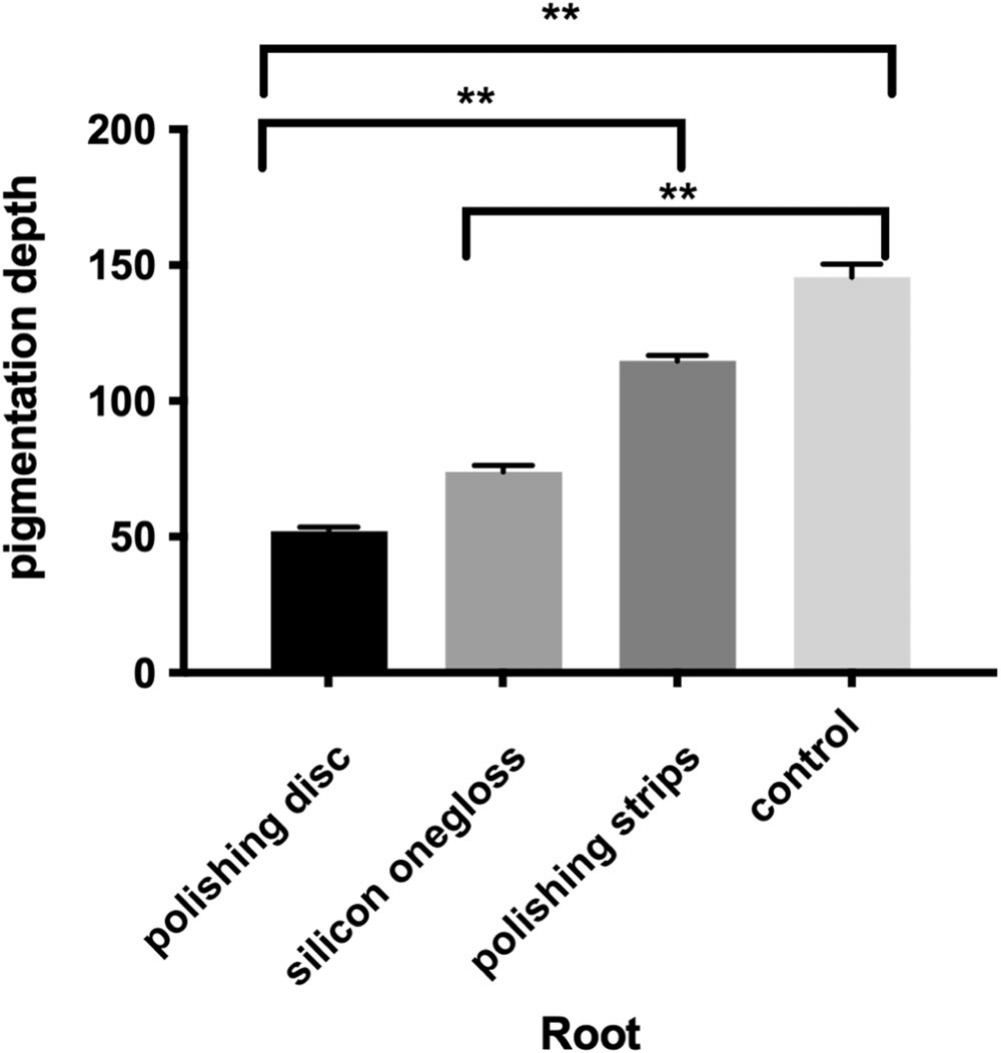
Roots assessed by laser scanning confocal microscopy.

The results of confocal microscopy are shown Figures 8, 9 and Tables 7, 8). According to the coronal results, there was no significant difference in the pigmentation depth between the polishing disc group and the silicon onegloss group, and there was no significant difference between the polishing strip group and the control group; however, the depths of the polishing disc group and the silicon onegloss group were significantly lower than those of the polishing strip group and the control group (Tables 9 and 10). In terms of the root area, the depth in the polishing disc group was significantly lower than that of the polishing strip group and control group, and the depth in the silicon onegloss group was significantly lower than that of the control group. There were no other significant difference in the pigmentation depth among the four groups (Figures 10 and 11). In addition, the decrease in pigmentation depth after root polishing was greater than that after crown polishing within each polishing system group (Table 11).

**Table 7:** Pigmentation depth (μm) of the three different polishing methods and the control group.

	Crown
Polishing disc	24.1387 ± 1.0581
Silicon onegloss	26.7080 ± 1.1745
Polishing strip	36.7893 ± 0.8607
Control	38.8880 ± 0.7423

**Table 8:** Pigmentation depth (μm) of the three different polishing methods and the control group.

	Root
Polishing disc	52.0107 ± 1.4906
Silicon onegloss	73.8893 ± 2.3882
Polishing strip	114.7307 ± 2.0666
Control	145.6553 ± 4.8206

**Table 9:** Comparison of the pigmentation depths among the three different polishing methods and the control group.

	Crown
Polishing disc versus silicon onegloss	0.067
Polishing disc versus polishing strip	0.000**
Polishing disc versus control	0.000**
Silicon onegloss versus polishing strip	0.000**
Silicon onegloss versus control	0.000**
Polishing strip versus control	0.133

**p < 0.01.

**Table 10:** Comparison of the pigmentation depths among the three different polishing methods and the control group.

	Root
Polishing disc versus silicon onegloss	0.140
Polishing disc versus polishing strip	0.000**
Polishing disc versus control	0.000**
Silicon onegloss versus polishing strip	0.059
Silicon onegloss versus control	0.000**
Polishing strip versus control	0.289

**p < 0.01.

**Table 11:** Statistical detection of comparison of pigmentation depth between roots and crowns in the different experimental groups.

	Crown versus root
Polishing disc	0.000**
Silicon onegloss	0.000**
Polishing strip	0.000**

**p < 0.01.

### 3.2 Clinical trials

#### 3.2.1 Plaque staining

**Figure 12:**
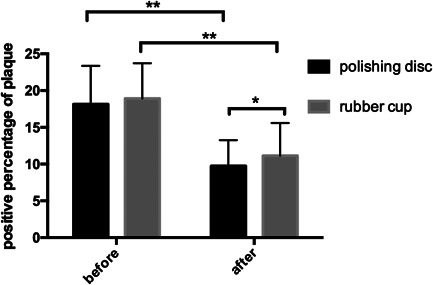
Positive percentage (%) of plaque staining before and after polishing with the rubber cup and polishing disc.

The plaque staining results showed that the positive percentage of plaque staining was approximately 18% after supragingival scaling and sandblasting, and there was no significant difference between the two groups (Table 12). After polishing with the rubber cup, the positive percentage of plaque staining was reduced to 11% (*p* = .000), and after polishing with the polishing disc, the positive percentage was reduced to 9% (*p* = .000). The decrease in VAS score in the experimental group was greater than that of the control group (*p* = .02) (Figure 12). This trend was similar to the results of LSCM, which indicated that polishing discs could reduce the adhesion of pigments and bacteria not only in vitro but also in vivo.

**Table 12:** Positive percentage (%) of plaque staining before and after polishing with the rubber cup and polishing disc.

	Before	After
Polishing disc	18.16 ± 5.20	9.74 ± 3.52
Rubber cup	18.93 ± 4.78	11.14 ± 4.46

#### 3.2.2 Patient VAS

**Figure 13:**
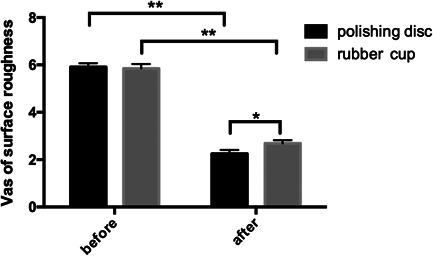
Surface roughness of the teeth before and after polishing with a polishing disc and a rubber cup.

The average VAS score for the roughness of the teeth before polishing was 5.91 in the experimental group and 5.85 in the control group. Moreover, there was no significant difference between the two groups. After polishing with a rubber cup, the average VAS score indicated that the roughness of the teeth in the control group decreased to 2.69 and that in the experimental group decreased to 2.25 (Table 13). Compared with that before polishing, the average VAS score after polishing was significantly lower in both groups (*P* Cup = 0.000, *P* disc = 0.000), and the decrease in the experimental group was greater than that of the control group (*p* = .042) (Figure 13). This may have been due to the results of the roughness measurement, which implied that polishing disc could reduce roughness by 25% (crowns) to 40% (roots) in vitro.

**Table 13:** Surface roughness of the teeth before and after polishing with a polishing disc and a rubber cup.

	Before	After
Polishing disc	5.91 ± 0.16	(*)2.25 ± 016(*)
Rubber cup	5.85 ± 0.19	(*)2.69 ± 0.13

*p < 0.05.

**Figure 14:**
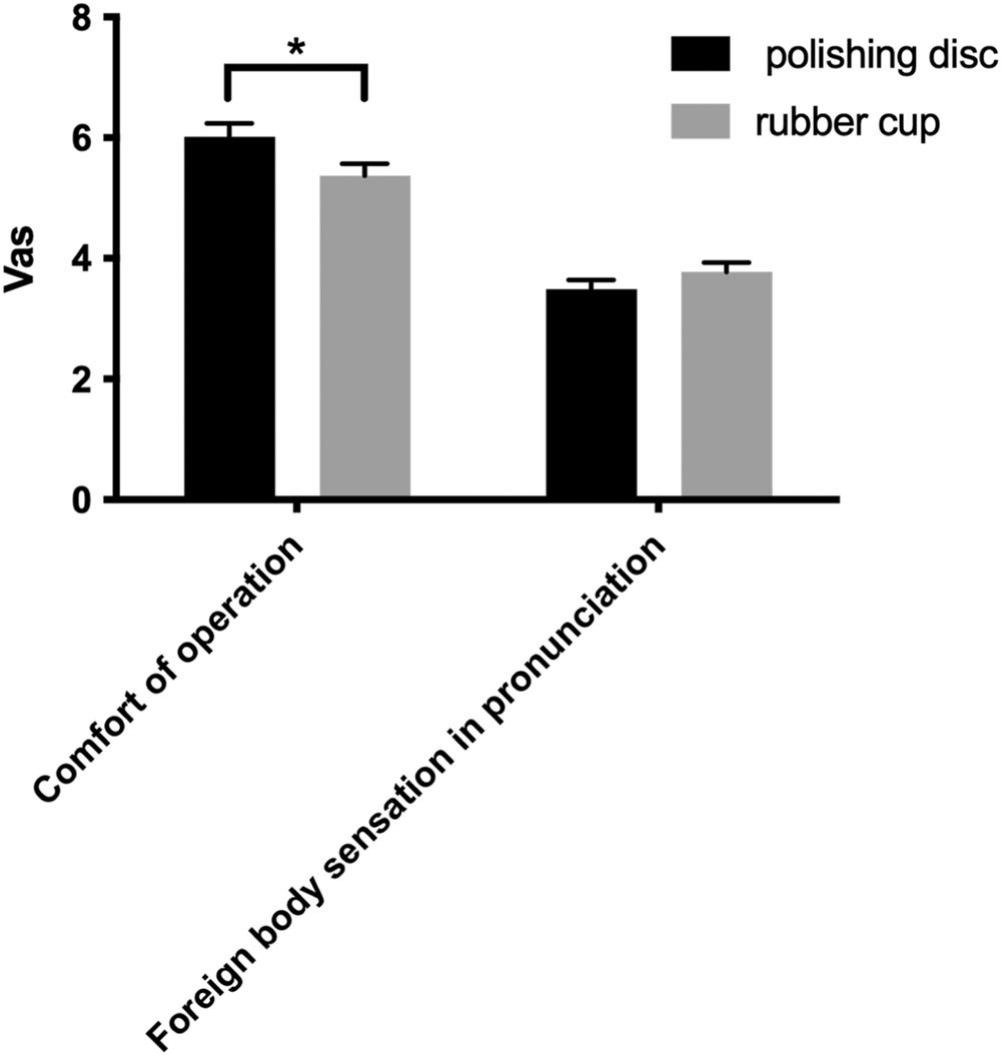
Comfort of operation and foreign body sensation in pronunciation after polishing with a polishing disc and a rubber cup.

Regarding the comfort of operation, the average VAS score was 6.02 in the experimental group and 5.37 in the control group, with the former being significantly greater than the latter (*p* = .038) (Table 14). There were no significant differences between the experimental group (mean value of 3.49) and the control group (mean value of 3.78) in terms of foreign body sensation during pronunciation (*p* = .178) (Figure 14).

**Table 14:** Comfort of operation and foreign body sensation in pronunciation after polishing with a polishing disc and a rubber cup.

	Comfort of operation	Foreign body sensation in pronunciation
Polishing disc	6.02 ± 0.22	3.49 ± 0.15
Rubber cup	5.37 ± 0.20(*)	3.78 ± 0.15

*p < 0.05.

## 4 DISCUSSION

In this study, the effects of different polishing methods were evaluated via in vitro testing to determine the most suitable method for further case verification to provide a reference and basis for clinicians to choose a polishing method.

Previous studies have shown that the combined use of ultrasonic instruments and manual instruments on the surface of teeth with periodontitis debridement can cause a certain degree of damage to the dentin and cementum of the teeth (DelPriore et al., 2023). Petersilka et al. (2003) reported that the number of subgingival bacteria was significantly greater in periodontally affected teeth treated with scaling and curettage than in periodontally affected teeth treated with polishing after scaling and curettage. While paying attention to the adverse effects of scaling and curettage on the tooth surface, some studies have found that sandblasting has adverse effects on the tooth surface. By sandblasting the root of the teeth in vitro, Fang and Xiaoping (2011) reported that the surface roughness of the cementum was significantly reduced after polishing. These findings are in line with those of other studies, who noted that sodium bicarbonate, which is the main component of sand commonly used in sandblasting, has a Mohs hardness of approximately 2.5, which is close to the Mohs hardness of cementum (Bühler et al., 2015). The sodium bicarbonate particle shape is irregular, and the edge is sharp (Comba et al., 2022), which roughens the particle surface. These factors all exacerbate the wear of cementum. Jana et al. (2016) used sandblasted particles of different sizes and compositions to polish teeth in vitro after scaling and finally found that only 3–5 μm hydroxyapatite crystals could decrease the surface roughness of sandblasted teeth reach Ra = 0.2 μm. For other particles, the size needed to be less than 1 µm to achieve the desired postblast roughness (Jana et al., 2016). Therefore, polishing treatment after basic therapy is an important factor for determining the prognostic effect of periodontal treatment.

To date, the main dental polishing instrument used in clinical practice is the rubber cup, which is usually paired with polishing paste. Several studies have shown that polishing with a rubber cup and polishing paste can significantly reduce enamel roughness after scaling (Mensi et al., 2022). For the root surface after manual curettage, the rubber cup can markedly reduce the instrument scratches on the surface, thus improving the surface smoothness. However, some studies have noted that the hollow shape of the rubber cup can easily lead to partial omission of the polishing area, and splashing of the polishing paste can occur during polishing, thus modifying the therapeutic effects on the patients (Wadia, 2021). To avoid the disadvantages above, some other methods can be tried to improve the polishing effect and patient satisfaction.

Silicon onegloss are made of high‐density aluminum oxide and silicone rubber. Because silicon contains polishing onegloss, it does not need to be used with polishing paste. Some studies have shown that silica onegloss positively affects the removal of orthodontic bracket adhesives, the polishing of fillings and the polishing of enamel. In this study, silicon onegloss obviously improved the surface morphology and roughness of the root of the isolated teeth, but there could be excessive cutting of the root cementum, coinciding with the results of other researchers (Ren et al., 2018). Therefore, further experimental and clinical studies would be needed in patients with root exposure.

Conventional polishing discs are usually made of a paper or plastic substrate, which is attached to the polishing particle material, metal core, and shaft rod. These discs are prone to breakage due to the substrate material. In addition, during polishing, the metal core can touch the tooth surface, causing tooth staining or defects. The SHOFU polishing disc (L507/522/519/521) is made of a soft and elastic material, which ensures that it does not break easily during polishing, and the plastic core is connected to the shaft rod to avoid damage to the tooth. In addition, the disc has a brand new 3D spherical abrasive coating, which enhances the removal of debris during polishing and resulting in a smooth, shiny surface with relatively low heat generation (Tepe et al., 2023). Some studies have shown that the surface roughness of orthodontic teeth after bracket removal can be significantly reduced by using state‐of‐the‐art polishing discs (Fan et al., 2017; Vadavadagi et al., 2019). These findings are in agreement with the results of our study.

Polishing strips are made by gluing bauxite particles, which is an artificial abrasive material, onto one side of a strip of polyester film. The strips are easy to use without the requirement of a low‐speed dental handpiece and are less likely to damage teeth and gums. However, due to the low efficiency of polishing, the strips are generally suitable for adjacent areas that low‐speed dental handpieces could not touch. Some scholars have shown that the strips could be used to polish the surfaces of adjacent fillings, and the surface roughness and patient comfort would significantly improve (Ozdemir et al., 2022).

Plaque in periodontitis patients are generally divided into supragingival and subgingival plaques according to the location of distribution. The plaque on the gingival surface is generally distributed in the enamel of the cemento–enamel junction or gingival margin and mainly consists of Gram‐positive cocci and bacilli. Subgingival plaque is generally distributed in the cementum around the root of the cemento–enamel junction in the periodontal pocket and mainly consisted of Gram‐negative bacilli. In dental plaque, there are multiple microenvironments with different pH values and concentrations, enabling various species with different metabolic needs to survive in them, thus increasing the diversity of bacterial populations. Therefore, there is a need for different research classifications based on different plaque distributions. In addition, enamel is the main surface tissue in the crown of the cemento–enamel junction, which exhibits increased hardness and wear resistance. The cementum is the main surface tissue at the root of the cemento–enamel junction; additionally, its compositional structure is looser and its hardness is much lower than that of the enamel. Because of the existence of root furcation in premolars and molars, basic treatment and polishing are insufficient once the furcation area is pathologically involved. Therefore, the average height of the root bifurcation limits the scope of periodontal treatment and polishing (Wang et al., 2023). Therefore, it would be more suitable in clinical practice to limit the range of tooth surface detection from 5 mm in the crown to 5 mm in the root with the cemento–enamel junction as the midline, which has a certain reference value.

The results of the in vitro tooth roughness test showed that the crown polishing effect of each group was weaker than that of root polishing, which could be because the initial roughness of the roots was greater than that of the crown, and the hardness of the root cementum was lower than that of the crown enamel (Yucesoy et al., 2023). According to the crown polishing results, the effects of the silicon onegloss group and the polishing disc group were similar and better than those of the polishing strip group. These results could have occurred because although the crown shape was smooth and the contact areas resulting from the different polishing methods were similar, the rotation speeds of the silicon onegloss and polishing discs were better than those of the polishing strips. Considering the polishing effect on the root side, the polishing disc treatment was better than the silicon particle treatment, which in turn was better than the polishing strip treatment. This phenomenon could have occurred due to the irregular shape of the root surface. Moreover, the silicon onegloss would produce a greater cutting effect under pressure, resulting in root surface cementum wear (Gaikwad & Sokolov, 2008). The polishing disc had a soft texture and high elasticity, and its cutting effect was far less than that of silicon onegloss. Confocal scanning was used to measure the depth of the simulated surface colouration after polishing to further verify the decrease in surface roughness after different polishing methods. According to the crown and root results, the staining depths in the polishing disc group and silicon onegloss group were not significantly different. The staining depth of the teeth in both the polishing disc group and the silicon onegloss group was significantly lower than that of the polishing strip group and the control group. These findings were consistent with the results of the roughness test. In vitro experiments on root could estimate the subgingival polishing effect in clinic, which could compensate for the aspects that could not be evaluated in the clinical part. Therefore, we selected the polishing disc as a relatively good polishing method and the commonly used method as the rubber cup for further comparison.

As the initial factor of periodontal disease, plaque microbes should be controlled. Plaque staining directly indicates plaque attachment on the surface of the teeth. In general, a plaque percentage below 20% could be considered the basic control of plaque, and plaque percentages less than or equal to 10% indicate that the oral environment is adequate (Song & Ge, 2019). According to the experimental results, the plaque morphology of unpolished teeth could be basically controlled by scaling and sandblasting. After polishing with a rubber cup, there was obvious improvement, and after polishing with a polishing disc, a good plaque control target was achieved.

The VAS results showed that the effect of the polishing disc was similar to that of the rubber cup concerning the oral foreign body sensation. Considering the comfort of the operation and the roughness of the tooth surface, the effect of the polishing disc was obviously better than that of the rubber cup. All these results indicated that polishing with a polishing disc as a basic treatment could obviously improve the effects of treatment and the feelings of patients.

In summary, polishing with a polishing disc can obviously improve the roughness of the teeth and the clinical comfort of the patients after basic periodontal treatment, and the polishing disc could replace the rubber cup to polish the teeth after basic periodontal treatment. More options could be made for clinicians when choosing a polishing method, but long‐term results must be confirmed in additional clinical trials.

## AUTHOR CONTRIBUTIONS

Jingjue Gong contributed to conception, design, data acquisition and interpretation, performed all statistical analyses, drafted and critically revised the manuscript. Xin Huang contributed to conception, design, and critically revised the manuscript. Shuang Yuan contributed to conception, design, and critically revised the manuscript. All authors gave their final approval and agreed to be accountable for all aspects of the work.

## CONFLICT OF INTEREST STATEMENT

The authors declare no conflicts of interest.

## Data Availability

The authors confirm that the data supporting the findings of this study are available within the article.
